# Individualized home-based exercise and nutrition interventions improve frailty in older adults: a randomized controlled trial

**DOI:** 10.1186/s12966-019-0855-9

**Published:** 2019-12-02

**Authors:** Tsung-Jen Hsieh, Shin-Chang Su, Chun-Wei Chen, Yaw-Wen Kang, Ming-Hsia Hu, Li-Lin Hsu, Szu-Yun Wu, Likwang Chen, Hsing-Yi Chang, Shao-Yuan Chuang, Wen-Harn Pan, Chih-Cheng Hsu

**Affiliations:** 1 Institute of Population Health Sciences, National Health Research Institutes, 35 Keyan Road, Zhunan, Miaoli County, 35053 Taiwan; 2 School and Graduate Institute of Physical Therapy, College of Medicine, National Taiwan University, Taipei, Taiwan; 3 Department of Chest Medicine, Miaoli General Hospital, Miaoli, Taiwan; 4 Department of Physical Medicine and Rehabilitation, Miaoli General Hospital, Miaoli, Taiwan; 5 Food Industry Research and Development Institute, Hsinchu, Taiwan; 6 Institute of Biomedical Sciences, Academia Sinica, 128 Academia Road, Section 2, Nankang, Taipei, 11529 Taiwan; 7 Department of Health Services Administration, China Medical University, Taichung, Taiwan; 8 Department of Family Medicine, Min-Sheng General Hospital, Taoyuan, Taiwan

**Keywords:** Frailty, Older adults, Exercise, Nutrition, Individualized home-based interventions

## Abstract

**Background:**

Frail older adults are predisposed to multiple comorbidities and adverse events. Recent interventional studies have shown that frailty can be improved and managed. In this study, effective individualized home-based exercise and nutrition interventions were developed for reducing frailty in older adults.

**Methods:**

This study was a four-arm, single-blind, randomized controlled trial conducted between October 2015 and June 2017 at Miaoli General Hospital in Taiwan. Overall, 319 pre-frail or frail older adults were randomly assigned into one of the four study groups (control, exercise, nutrition, and exercise plus nutrition [combination]) and followed up during a 3-month intervention period and 3-month self-maintenance period. Improvement in frailty scores was the primary outcome. Secondary outcomes included improvements in physical performance and mental health. The measurements were performed at baseline, 1 month, 3 months, and 6 months.

**Results:**

At the 6-month measurement, the exercise (difference in frailty score change from baseline: − 0.23; 95% confidence interval [CI]: − 0.41, − 0.05; *p* = 0.012), nutrition (− 0.28; 95% CI: − 0.46, − 0.11; *p* = 0.002), and combination (− 0.34; 95% CI: − 0.52, − 0.16; *p* <  0.001) groups exhibited significantly greater improvements in the frailty scores than the control group. Significant improvements were also observed in several physical performance parameters in the exercise, nutrition, and combination groups, as well as in the 12-Item Short Form Health Survey mental component summary score for the nutrition group.

**Conclusions:**

The designated home-based exercise and nutrition interventions can help pre-frail or frail older adults to improve their frailty score and physical performance.

**Trial registration:**

Retrospectively registered at ClinicalTrials.gov (identifier: NCT03477097); registration date: March 26, 2018.

## Introduction

Frailty is a complicated geriatric syndrome characterized by low physiological reserves and decreased resistance to stressor events. The causes of frailty are multifactorial, including genetic, environmental, physical, and nutritional factors [1]. Frailty is considered an age-related deficiency in both the physiological and psychological domains [2]. The results from a systematic review reported that the prevalence of frailty increased with age [3]. With rapid and tremendous growth of the older population over the past two decades, frailty deserves special attention. Conceptualization of the design and execution of effective frailty prevention and management protocols are necessary because frail people have high risks of adverse health outcomes, such as functional disability, hospitalization, and death [1, 4].

Our previous study determined that older adults with a dietary pattern with large amounts phytonutrient-rich plant foods and protein-rich foods had a lower risk of frailty than their counterparts did [5]. Recent studies have also shown that frailty can be improved and managed through suitable interventions [6, 7]. Exercise and nutrition interventions are two major non-pharmacological approaches that are effective in improving muscle mass, functional ability (mobility and activities of daily living), fitness levels, and cognitive functions [8–10]. Because frailty is comprised of multiple correlated functional declines, multicomponent interventions for frailty prevention has received attention [7, 11]. However, most interventional programs are conducted at centers or designed as group activities; consequently, the programs are less accessible to older adults who are not willing to travel regularly to these centers. Hence, the development of home-based, self-practiced, multicomponent intervention protocols for managing frailty in outpatient facilities is advisable.

To our knowledge, studies on home-based multicomponent interventions for frailty syndrome management are still limited. Therefore, in this study, individualized home-based exercise and nutrition interventions were developed to assist older adults who seek clinical help. We hypothesized that this intervention program is efficient in improving not only the frailty scores but also related physical and mental health outcomes for pre-frail or frail older adults.

## Methods

### Study design and participants

This study was a four-arm, single-blind, randomized controlled trial that was conducted between October 2015 and June 2017 in the outpatient clinics of Miaoli General Hospital in Taiwan and registered at ClinicalTrials.gov (NCT03477097). The Institutional Review Board on Biomedical Science Research of Academia Sinica in Taiwan approved the protocol, informed consent form, and case report forms (AS-IRB01–15020). All participants signed the informed consent forms. This study followed the CONSORT guidelines for reporting [12].

In outpatient clinics, individuals aged ≥65 years of age were invited to participate in this study. Trained case managers screened the frail or pre-frail older adults by using the Cardiovascular Health Study Criteria [4]. Participants who were non-frail, were unable to walk a 14-m distance independently; had severe illnesses (e.g., cancer), severe depression (Geriatric Depression Scale [GDS] [13] ≥ 10), cognitive impairment (Mini-Mental State Examination [MMSE] score [14] < 24 for the literate older adults or < 18 for the illiterate); had communication impairment, were hospitalized or were living in a nursing home, had participated in other studies; or were taking nutritional supplements at the time of recruitment were excluded.

The trained case managers evaluated older adults for eligibility by using a comprehensive geriatric assessment and physical performance tests (e.g., handgrip strength, gait speed). Those who were eligible and provided informed consent were randomly assigned into one of the four study groups: control, exercise, nutrition, and combination (exercise plus nutrition), each with 3-month intervention and 3-month self-maintenance periods. The permuted-block randomization (12 per block) procedure was used. They were then referred to licensed physiotherapists or dietitians for further individualized evaluation and intervention.

### Intervention contents

The exercise intervention consisted of a combination of strength, flexibility, balance, and endurance training based on the guidelines of the American College of Sports Medicine [15]. At the outset, the trained case managers evaluated the physical fitness of each participant based on multiple facets (handgrip strength, gait speed, upper and lower body flexibility, lower extremity strength, balance and leg strength, and volume of physical activity). In order to enhance participants’ fitness levels, the participants received personalized (fitted to individual needs) exercise prescriptions and handy tools (e.g., resistance band, grip-ball, and pedometer) from the licensed physiotherapists. Approximately 3 to 7 exercise sessions per week were recommended, with the time (5 to 60 min) per session or repetitions tailored to participants’ capabilities. Each participant in the exercise and combination groups was encouraged to perform a 6-month home-based training. The performance of daily exercise was recorded in an exercise diary.

The underlying concept of nutrition intervention was to maintain a desirable body weight by maintaining an appropriate level of caloric intake achieved through a designated number of servings (as suggested by the Taiwanese Food Guide [16]) of six food groups (dairy; protein-rich foods; vegetables; fruits; nuts, seeds, plant oils; and grains or roots). Caloric requirements were appraised considering the age, sex, height, weight, and physical activity levels of the participants. The participants received a set of customized dishware, including a plate with four compartments for vegetables and protein foods, a bowl for rice and fruits, a mug for milk and juice, and a tablespoon. A colored meal pad was given to indicate the personalized food amount on the dishware, which was designed to help participants easily obtain the correct amounts of rice, protein foods, fruits, vegetables, milk, and nuts or seeds. The detailed description of this dietary intervention method and customized dishware was as previously published [17]. Moreover, participants in the nutrition and combination groups were asked to record the number of servings they consumed from each of the six food groups (bowl for rice, mug for milk, compartment for protein foods and vegetables, fist-size for fruits, and a tablespoon for nuts or seeds).

In addition, the following two food supplements were provided: 25 g of skim milk powder a day and 10 g of mixed nuts a day (cashews, almonds, pumpkin seeds, walnuts, macadamia nuts, and pine nuts) (nutrition-1 subgroup).

Oxidative stress of aging has been associated with sarcopenia, and no clear evidence exists in the literature regarding the protective effect of antioxidant supplementation [18]. In addition, the potential of fish oil to manage anabolic resistance to protein in sarcopenia is well known [19]. Therefore, we randomly provided half of the participants in the nutrition and combination groups additional nutritional supplements, including three fish oil capsules and one vegetable and fruit concentrate capsule per day (nutrition-2 subgroup). Each fish oil capsule (500 mg/capsule) contained 140 mg of eicosapentaenoic acid and 95 mg of docosahexaenoic acid (DSM MEG-3™ 3322EE Oil). Each 200 mg vegetable and fruit capsule contained water- and ethanol-extracted vegetable and fruit concentrate (Oxxynea® FP) with an anti-oxidative potential equivalent to four servings of fruits and vegetables. However, because the fish oil and vegetable and fruit concentrate did not exert any additional effect in this trial, we present the overall findings of the nutrition intervention.

### Patient management

All participants were contacted by telephone by case managers on the third day after the first intervention. After this, participants followed the following schedule: visiting case managers, physiotherapists, or dietitians in person at the end of the first month, receiving a telephone call at the end of the second month, and making additional visits in person to case managers, physiotherapists, or dietitians at the end of the third and sixth months. Moreover, the participants in the intervention groups received inspirational cards at the 1-month and 3-month follow-ups, encouraging them to maintain their designated intervention schedules.

The physiotherapists and dietitians called and encouraged the subjects in the exercise and nutrition arms on the third day and in the second month. At the end of 1-month and 3-month follow-ups, physiotherapists and dieticians examined the exercise and dietary diaries of each participant. The interventions were modified to suit participants’ individual needs if participants did not achieve the desired amount of exercise or target dietary goals because of health-related reasons (e.g., muscle pain, decrease in appetite, weight loss, or weight gain of more than 5%) in the preceding months. The physiotherapists and dietitians also encouraged the participants to maintain their exercise and balanced diet practices during the next 4–6 months.

As mentioned, participants in the combination group received both exercise and nutrition interventions, and those in the control group underwent regular medical care without any interventions except telephone contacts (for greeting only) by case managers on the third day and at the end of the second month.

### Dietary compliance

The compliance with dietary consultation was assessed. Using the dietary recall method and aids, such as food models and measuring dishware, licensed dietitians assessed the dietary intake within the preceding month by inquiring about the participants’ typical dietary patterns, most frequently consumed items, and the amount of these foods eaten at breakfast, lunch, dinner, and snack times. If a participant had several types of meals, drinks, or snack patterns at these time points, all were documented and weighted by the consumption probability (estimated from the frequency) to obtain an average intake profile. In addition, the discrepancies between recall and diary were clarified. The dietary intake data were transformed into nutrient data, including total calories, protein, carbohydrate, and fat, by using a computerized worksheet based on the Taiwan Food Nutrient Database. The method to estimate serving numbers of the six food groups is provided in the Appendix.

For compliance, we checked and tested the changes in protein, fat, and carbohydrate (for both weight [g/day] and percentage calorie contribution [%]) and changes in serving numbers of the six food groups per the nutrition intervention and exercise intervention statuses because the interaction between the nutrition and exercise interventions was not significant.

### Assessment of frailty and other measurements

The single-blind assessments of all participants were conducted at baseline, 1 month, 3 months, and 6 months by trained case managers who were unaware of which intervention group participants belonged.

The primary outcome of this study was the frailty score, which was quantified based on the Cardiovascular Health Study Criteria developed by Fried and colleagues [4]. The criteria comprise five components: unintentional weight loss, exhaustion, poor muscle strength, slowness, and low physical activity. Unintentional weight loss was defined as involuntary loss of > 3 kg (or 5%) of body weight in the preceding year. Exhaustion was assessed by responses to the following question: “How often in the last week did you feel that you could not get going?” If participants’ response indicated more than 3 days, they were considered positive for the exhaustion component. Poor muscle strength and slowness were respectively assessed based on handgrip strength and gait speed. Handgrip strength was evaluated using a standard hydraulic hand dynamometer (Baseline®, Fabrication Enterprises, Inc., NY, USA), and gait speed was evaluated using a 10-m walk test with 2 m added at the beginning and end of the pathway to eliminate the effects of acceleration and deceleration. The sex- and body mass index-specific cut-off points and the sex- and height-specific cut-off points were used to identify low handgrip strength and slow gait speed, respectively [20]. Moreover, physical activity was evaluated using the Taiwan International Physical Activity Questionnaire (IPAQ)–Short Form for the Elderly (Taiwanese version of IPAQ [21], plus an additional assessment of light activity); sex-specific cut-off points were employed to assess low physical activity [22]. For the five frailty criteria, a score of 1 was given if a criterion was met. The total scores ranged between 0 and 5, and participants would be classified as frailty (three or more scores), pre-frailty (one or two scores), or robustness states (zero score).

The secondary outcomes of this study included evaluations of physical performance and mental health status. For physical performance, we measured handgrip strength, gait speed, upper body flexibility (back scratch) [23], lower body flexibility (chair sit-and-reach) [23], and lower extremity strength (standing heel-rise) [24]. The mental health outcomes composed of the GDS and 12-Item Short Form Health Survey mental component summary (SF-12 MCS) scores [25].

### Sample size calculation

The previous study had reported that the significant difference in the frailty score between the exercise and control groups was 0.23 after the 6-month intervention [6]. We postulated that exercise plus nutrition intervention would offer a better reduction in the frailty score than the exercise intervention alone. Accordingly, sample size calculation was based on an assumed reduction in the frailty score of 0.3 points between the three intervention groups and the control group. We assumed that the correlations among repeated measures were 0.2. The requirement of minimum sample size in each group was 66 participants to reach the statistical significance at an overall significance level of 0.05 and a power of 80%. In addition, the total sample size was 320 by assuming a 20% attrition during follow-up. The sample size calculation was performed using the G*Power analysis program [26].

### Statistical analysis

The participants’ baseline demographics and health-related characteristics among the designated groups were expressed as mean ± standard deviation (SD) or number (percentage) for continuous variables or categorical variables, respectively. The Kruskal–Wallis test, chi-square test, and Fisher’s exact test were performed to assess the differences in baseline measurements among the designated groups. This trial was analyzed based on the intention-to-treat (ITT) principle [12], and the last observation carried forward method was utilized to impute missing values owing to participants dropping out or being lost to follow-up. Comparisons of intervention effects over time on measured outcomes were implemented using the linear mixed-effects models. In the models, “changes from baseline” in frailty score and secondary outcomes were considered response variables; explanatory variables included the intervention groups, time effect, and interaction between the intervention groups and time effect. In addition, to consider correlations among the repeated measures for each participant, an autoregressive correlation structure was used, which assumed that successive measures correlated more highly than non-successive measures for the same participant. The statistical significance of all tests was evaluated at a predetermined significance level of 0.05, and post hoc tests between the three intervention groups and the control group were assessed at an adjusted significance level of 0.017 by using the Bonferroni correction [27]. All data analyses were carried out using the SAS statistical software version 9.4 (SAS Institute Inc., Cary, NC, USA).

## Results

**Fig. 1:**
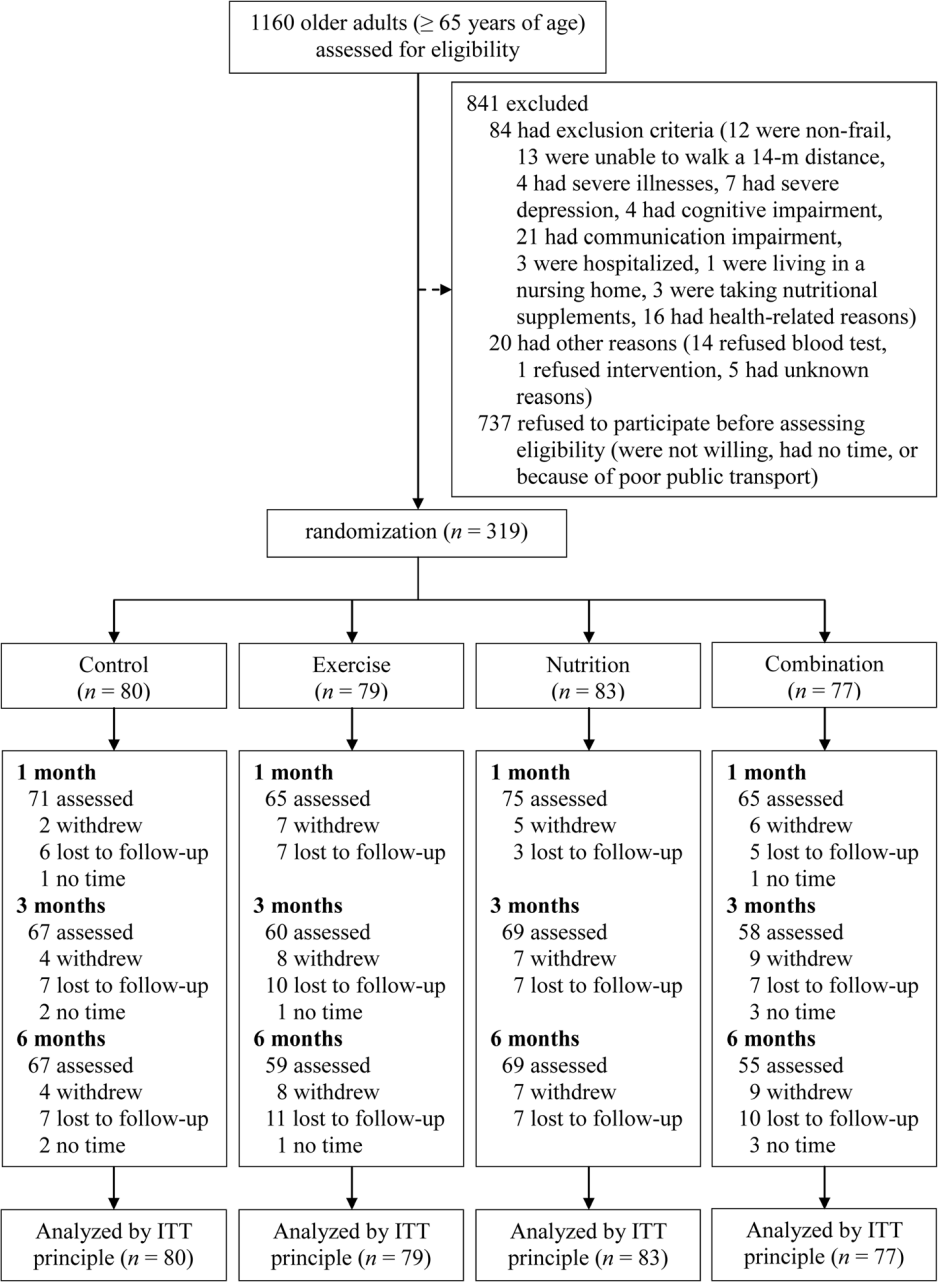
Flow chart of study participants in the frailty intervention randomized controlled trial. Legend: ITT, intention-to-treat

Among the 1160 invited older adults, 737 (63.6%) refused to participate before assessing eligibility, 84 (7.2%) were ineligible, 20 eligible subjects (1.7%) dropped out in the run-in period, and an overall 319 (27.5%) eligible subjects agreed to participate and were randomized into four designated groups (as shown in Fig. 1). The compliance rates of follow-up assessment were 87, 80, and 78% for the 1-month, 3-month, and 6-month follow-ups, respectively. The mean age of study participants was 71.6 ± 5.7 years, and 39.8% of the participants were women. The prevalence rates of pre-frail older adults among the four designated groups were within 86.1–93.5% and the prevalence rates of frail older adults among the four designated groups were within 6.5–13.9%. Baseline demographics and health-related characteristics of the study participants in the four designated groups are shown in Table 1. No statistically significant differences were observed in most variables, except for lower body flexibility, lower extremity strength, and the prevalence rate of diabetes mellitus.

**Table 1:** Baseline demographics and health-related characteristics of participants in the four designated groups

Characteristics	Total *n*^a^	Control (*n* = 80)	Exercise (*n* = 79)	Nutrition (*n* = 83)	Combination (*n* = 77)	*p*-values
Age (years)	319	72.5 ± 5.5	72.0 ± 6.0	70.4 ± 5.3	71.6 ± 6.0	0.101
Sex (women)	319	29 (36.3)	33 (41.8)	38 (45.8)	27 (35.1)	0.474
Education (> 6 years)	319	45 (56.3)	42 (53.2)	45 (54.2)	44 (57.1)	0.957
ADL dependency (< 60 points)	319	0 (0.0)	0 (0.0)	1 (1.2)	1 (1.3)	0.740
GDS (scores)	319	2.2 ± 1.7	2.5 ± 1.8	2.6 ± 1.9	2.4 ± 1.6	0.441
SF-12 MCS (scores)	318	53.8 ± 7.9	53.9 ± 8.1	53.4 ± 7.8	55.8 ± 7.0	0.404
MMSE (scores)	319	27.7 ± 1.9	27.6 ± 2.3	27.4 ± 2.2	27.6 ± 2.4	0.781
BMI (kg/m^2^)	319	25.1 ± 3.5	25.1 ± 3.3	25.5 ± 3.4	24.4 ± 3.7	0.293
Handgrip strength (kg)	318	25.0 ± 7.8	24.5 ± 7.7	24.9 ± 9.2	26.9 ± 8.5	0.275
10-m gait speed (seconds)	319	10. 7 ± 3.2	12.5 ± 7.7	13.0 ± 7.4	11.4 ± 4.3	0.103
Upper body flexibility (cm)	298	−13.9 ± 12.3	− 12.9 ± 16.7	−13.2 ± 12.0	− 12.4 ± 12.5	0.699
Lower body flexibility (cm)	304	−4.6 ± 8.9	− 2.9 ± 8.5	−5.2 ± 8.1	−3.3 ± 7.6	0.015
Lower extremity strength (number)	264	1.6 ± 3.0	3.9 ± 5.2	2.0 ± 3.3	3.8 ± 4.3	< 0.001
Frailty score	319	1.5 ± 0.6	1.6 ± 0.8	1.7 ± 0.8	1.5 ± 0.7	0.687
Frailty status	319					0.287
Pre-frail		74 (92.5)	68 (86.1)	72 (86.7)	72 (93.5)	
Frail		6 (7.5)	11 (13.9)	11 (13.3)	5 (6.5)	
Frailty five indicators						
Weight loss ≥3 kg	318	27 (33.8)	23 (29.1)	24 (28.9)	21 (27.6)	0.846
Exhaustion	319	56 (70.0)	55 (69.6)	63 (75.9)	57 (74.0)	0.766
Poor muscle strength	318	26 (32.5)	28 (35.4)	23 (28.1)	21 (27.3)	0.649
Slowness	319	8 (10.0)	13 (16.5)	17 (20.5)	11 (14.3)	0.310
Low physical activity	316	4 (5.0)	9 (11.5)	11 (13.4)	7 (9.2)	0.309
Comorbidity						
Hypertension	318	52 (65.0)	51 (64.6)	58 (69.9)	56 (73.7)	0.567
Diabetes mellitus	318	28 (35.0)	39 (49.4)	24 (28.9)	20 (26.3)	0.012
Myocardial infraction	318	0 (0.0)	1 (1.3)	0 (0.0)	1 (1.3)	0.367
Stroke	318	7 (8.8)	13 (16.5)	6 (7.2)	6 (7.9)	0.183
Cancer	318	2 (2.5)	4 (5.1)	2 (2.4)	1 (1.3)	0.581

The data are expressed as mean ± SD or *n* (%)

*ADL* activities of daily living; *GDS* Geriatric Depression Scale; *SF-12 MCS* 12-Item Short Form Health Survey mental component summary; *MMSE* Mini-Mental State Examination; *BMI* body mass index

^a^Participants with available information

**Fig. 2:**
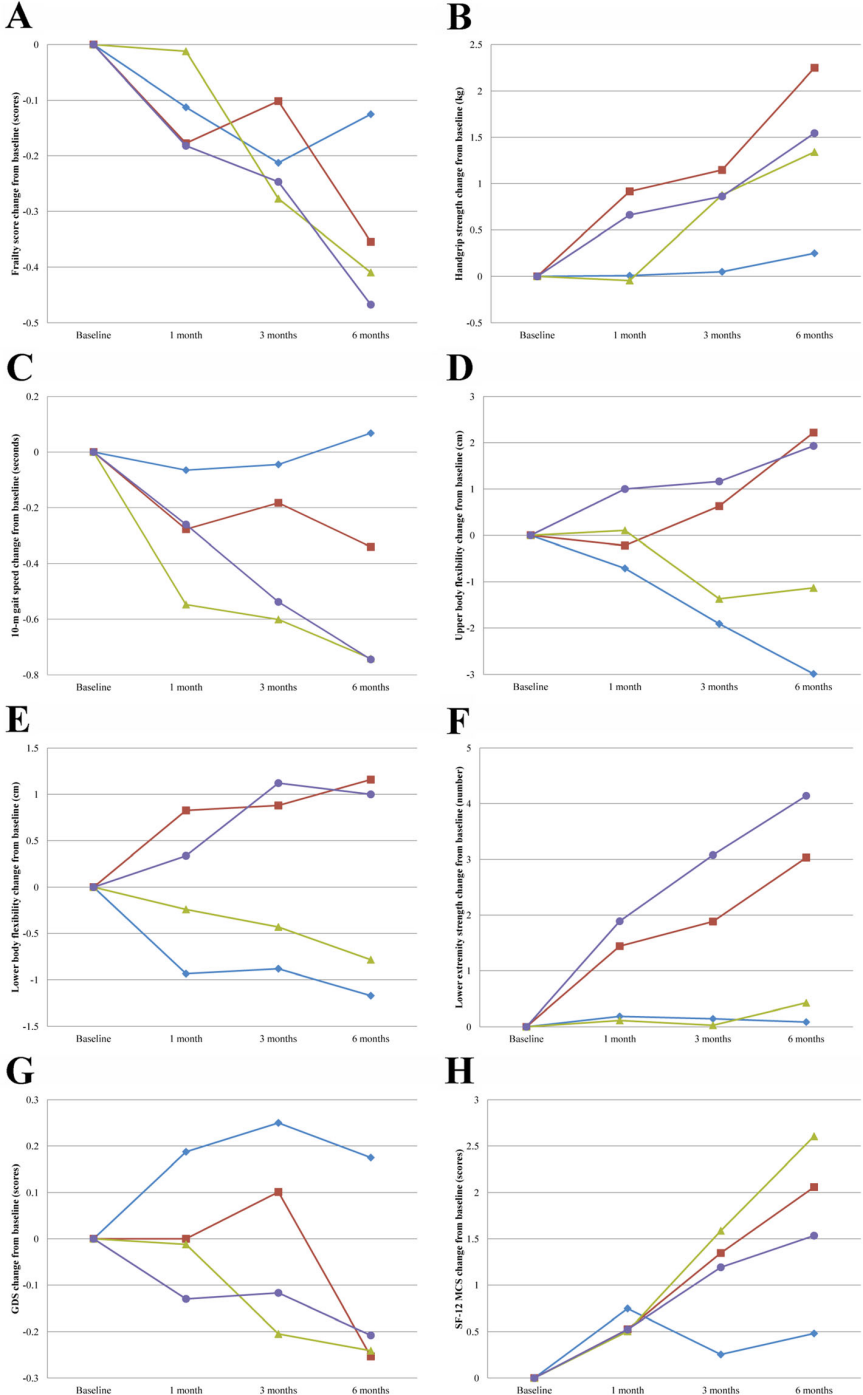
Mean changes from baseline in frailty score, physical performance, and mental health outcomes during the 6-month study period. Legend: **a**: Frailty score; **b**: Handgrip strength; **c**: 10-m gait speed; **d**: Upper body flexibility; **e**: Lower body flexibility; **f**: Lower extremity strength; **g**: Geriatric Depression Scale; **h**: 12-Item Short Form Health Survey mental component summary. : Control group; : Exercise group; : Nutrition group; : Combination group

Figure 2 depicts the mean changes from baseline for the primary outcome (frailty score) and secondary outcomes (handgrip strength, gait speed, upper body flexibility, lower body flexibility, lower extremity strength, GDS, and SF-12 MCS score) during the study period for the four designated groups. The changes of frailty score between baseline and follow-up assessments for the intervention and control groups are shown in Table 2. The significant time effect (*p* <  0.001) and the interaction effect between the intervention groups and time (*p* <  0.001) indicated that the group effects on the frailty score change from baseline differed significantly over time. Based on the results of the post hoc tests, the exercise (difference in frailty score change from baseline: − 0.23; 95% confidence interval [CI]: − 0.41, − 0.05; *p* = 0.012), nutrition (− 0.28; 95% CI: − 0.46, − 0.11; *p* = 0.002), and combination (− 0.34; 95% CI: − 0.52, − 0.16; *p* <  0.001) groups revealed statistically significant improvement in the frailty score after 6-month follow-up compared with the control group.

**Table 2:** Intervention effects on frailty score during the 6-month study period

		Mean ± SD				Difference in difference: change from baseline in each intervention group minus change from baseline in control group (95% CI)			*p* for groups × time interaction	*p* for groups	*p* for time
Outcome		Control	Exercise	Nutrition	Combination	Exercise	Nutrition	Combination			
Frailty score (scores)	*n*	80	79	83	77				< 0.001	0.402	< 0.001
	Baseline	1.5 ± 0.6	1.6 ± 0.8	1.7 ± 0.8	1.5 ± 0.7						
	1 month	1.4 ± 0.8	1.4 ± 0.8	1.7 ± 0.9	1.3 ± 0.8	−0.06 (−0.24, 0.11)	0.10 (− 0.08, 0.28)	− 0.07(− 0.25, 0.11)			
	3 months	1.3 ± 0.8	1.5 ± 0.9	1.4 ± 0.9	1.3 ± 0.7	0.11 (− 0.07, 0.29)	− 0.06 (− 0.24, 0.11)	− 0.03 (− 0.21, 0.15)			
	6 months	1.4 ± 0.9	1.3 ± 0.9	1.3 ± 0.9	1.1 ± 0.8	− 0.23 (− 0.41, − 0.05)^a^	−0.28 (− 0.46, − 0.11)^a^	−0.34 (− 0.52, − 0.16)^a^			

*CI* confidence interval; ^a^Pairwise comparisons (interventions vs. control) were significant at the post hoc significance level of 0.017

The results of the compliance with dietary consultation are presented in Table 3. We examined for any differences in the baseline mean or changes at 1 month, 3 months, and 6 months by exercise intervention status and by nutrition intervention status. Because of no interaction effects between the exercise and nutrition interventions, the statistical testing results of the exercise and nutrition intervention effects are presented separately. At baseline, no statistically significant differences were observed for both the exercise and nutrition intervention statuses for any of the dietary parameters that we examined. Notably, the nutrition intervention increased the intake levels of total calories, protein, carbohydrate, and fat at 1 month, 3 months, and 6 months. For investigation of energy from these macronutrients, the percentage of calories from protein increased significantly at all three follow-up time points in the group that received the nutrition intervention. No statistically significant changes for percent of calories were observed in carbohydrate and fat, except at 1 month (small decrease in carbohydrate and small increase in fat). This observation was because of the significant increases in the servings of dairy, beans/fish/meat/eggs, and oils/nuts. Vegetable consumption was approximately three servings a day at baseline, and no changes were seen. A significant, albeit modest, increase (less than half of serving) was observed for fruits.

**Table 3:** The changes of calorie, protein, carbohydrate, and fat, and changes in servings of the six food groups during the 6-month study period

Dietary compliance variables		Mean ± SD				*p* for main effects^a^	
		Control	Exercise	Nutrition	Combination	Exercise status^b^	Nutrition status^c^
	*n*	80	78	83	74		
Total energy (Kcal/day)	Baseline	1706.5 ± 435.8	1623.9 ± 480.6	1696.2 ± 485.4	1772.2 ± 462.2	0.945	0.210
	Change^d^ at 1 month	48.5 ± 294.3	13.7 ± 272.3	138.1 ± 324.5	186.2 ± 336.5	0.853	< 0.001
	Change at 3 months	52.5 ± 293.6	24.1 ± 311.3	170.1 ± 358.8	162.0 ± 311.2	0.612	< 0.001
	Change at 6 months	− 0.1 ± 277.2	−35.7 ± 342.8	102.1 ± 391.3	142.8 ± 327.1	0.951	< 0.001
Total protein (g/day)	Baseline	64.9 ± 18.6	60.8 ± 19.8	62.7 ± 18.2	64.8 ± 19.0	0.628	0.705
	Change at 1 month	−1.0 ± 14.1	0.4 ± 13.0	8.7 ± 18.8	9.6 ± 16.7	0.523	< 0.001
	Change at 3 months	− 0.2 ± 12.2	2.3 ± 12.1	9.2 ± 17.2	8.7 ± 16.3	0.549	< 0.001
	Change at 6 months	−2.2 ± 12.4	− 1.3 ± 13.0	7.2 ± 18.5	8.6 ± 16.9	0.521	< 0.001
Total protein (% in total energy)	Baseline	15.5 ± 2.7	14.9 ± 2.7	14.9 ± 2.4	14.8 ± 2.7	0.298	0.241
	Change at 1 month	− 0.4 ± 3.1	0.1 ± 2.5	0.6 ± 2.5	0.5 ± 2.3	0.556	0.017
	Change at 3 months	− 0.5 ± 2.6	0.5 ± 2.8	0.6 ± 2.2	0.5 ± 2.4	0.090	0.034
	Change at 6 months	− 0.4 ± 2.7	0.1 ± 2.8	0.8 ± 2.3	0.7 ± 2.4	0.484	0.003
Total carbohydrate (g/day)	Baseline	225.5 ± 64.6	215.7 ± 64.8	220.9 ± 60.9	229.5 ± 71.3	0.937	0.562
	Change at 1 month	8.7 ± 40.3	3.1 ± 43.4	16.2 ± 48.7	12.5 ± 42.9	0.351	0.089
	Change at 3 months	6.1 ± 35.4	0.9 ± 50.8	17.7 ± 46.9	13.1 ± 39.3	0.319	0.016
	Change at 6 months	0.7 ± 40.3	−5.7 ± 52.8	10.0 ± 44.9	13.6 ± 46.0	0.784	0.007
Total carbohydrate (% in total energy)	Baseline	53.7 ± 9.3	53.5 ± 8.4	52.6 ± 7.9	52.2 ± 10.0	0.774	0.253
	Change at 1 month	0.3 ± 7.2	− 0.1 ± 6.4	−0.9 ± 6.8	− 3.0 ± 6.9	0.105	0.010
	Change at 3 months	−0.7 ± 6.3	−1.0 ± 7.6	−1.6 ± 7.6	−2.2 ± 6.6	0.514	0.188
	Change at 6 months	0.2 ± 7.0	− 0.8 ± 7.7	−1.3 ± 7.5	−1.8 ± 7.2	0.333	0.124
Total fat (g/day)	Baseline	59.2 ± 26.3	58.7 ± 24.8	63.1 ± 27.9	65.9 ± 29.5	0.722	0.074
	Change at 1 month	1.8 ± 22.5	0 ± 16.6	5.2 ± 20.6	11.3 ± 23.1	0.361	0.002
	Change at 3 months	4.0 ± 22.1	1.1 ± 18.1	8.0 ± 24.3	8.5 ± 20.9	0.630	0.021
	Change at 6 months	−0.5 ± 21.5	−0.6 ± 20.4	4.7 ± 24.3	6.4 ± 21.0	0.738	0.015
Total fat (% in total energy)	Baseline	30.9 ± 8.9	31.6 ± 7.5	32.5 ± 8	33.0 ± 9.9	0.540	0.124
	Change at 1 month	0.1 ± 7.3	0 ± 6.1	0.3 ± 7.0	2.5 ± 7.0	0.166	0.095
	Change at 3 months	1.2 ± 6.6	0.5 ± 7.1	1.0 ± 7.6	1.7 ± 6.9	0.963	0.578
	Change at 6 months	0.2 ± 7.4	0.7 ± 7.5	0.5 ± 7.2	1.2 ± 7.5	0.473	0.633
Whole grains (bowls/day)	Baseline	2.9 ± 1.1	2.8 ± 1.0	2.8 ± 1.0	2.9 ± 1.0	0.989	0.844
	Change^d^ at 1 month	0.12 ± 0.7	0.05 ± 0.6	0.05 ± 0.8	0.02 ± 0.58	0.468	0.512
	Change at 3 months	0.02 ± 0.5	−0.04 ± 0.7	0.08 ± 0.7	0.02 ± 0.53	0.356	0.390
	Change at 6 months	− 0.05 ± 0.6	−0.13 ± 0.7	0.004 ± 0.6	0.04 ± 0.64	0.758	0.115
Beans/fish/meat/eggs (servings/day)	Baseline	5.2 ± 2.3	4.7 ± 2.3	4.6 ± 2.0	4.8 ± 1.9	0.534	0.252
	Change at 1 month	− 0.3 ± 2.2	− 0.1 ± 1.7	0.6 ± 2.2	0.7 ± 1.8	0.445	< 0.001
	Change at 3 months	−0.2 ± 1.7	0.1 ± 1.6	0.5 ± 1.8	0.6 ± 1.9	0.411	0.001
	Change at 6 months	−0.4 ± 1.7	−0.3 ± 1.8	0.5 ± 1.9	0.6 ± 1.8	0.612	< 0.001
Vegetables (servings/day)	Baseline	3.0 ± 1.6	2.9 ± 2.0	3.4 ± 1.9	3.4 ± 1.9	0.905	0.024
	Change at 1 month	0.2 ± 1.4	0.2 ± 1.3	0 ± 1.6	0.4 ± 1.4	0.233	0.753
	Change at 3 months	0.3 ± 1.3	0.3 ± 1.7	0.2 ± 1.6	0.3 ± 1.6	0.908	0.710
	Change at 6 months	0.2 ± 1.4	0.1 ± 1.9	0 ± 1.9	0.3 ± 1.6	0.589	0.936
Fruits (servings/day)	Baseline	1.8 ± 1.2	1.5 ± 0.9	1.7 ± 1.2	1.8 ± 1.5	0.460	0.325
	Change at 1 month	0 ± 1.1	0 ± 1.1	0.4 ± 1.2	0.4 ± 1.1	0.877	0.004
	Change at 3 months	0 ± 1.2	0.1 ± 1.1	0.3 ± 1.2	0.4 ± 1.2	0.475	0.035
	Change at 6 months	0.1 ± 1.2	0.2 ± 1.4	0.2 ± 1.2	0.5 ± 1.2	0.167	0.130
Dairy (mugs/day)	Baseline	0.3 ± 0.5	0.3 ± 0.4	0.4 ± 0.6	0.4 ± 0.5	0.805	0.231
	Change at 1 month	0 ± 0.3	0 ± 0.3	0.2 ± 0.6	0.2 ± 0.5	0.853	< 0.001
	Change at 3 months	0 ± 0.4	0.1 ± 0.3	0.3 ± 0.6	0.2 ± 0.5	0.522	< 0.001
	Change at 6 months	0.1 ± 0.4	0.1 ± 0.3	0.3 ± 0.6	0.1 ± 0.6	0.324	0.007
Oils/Nuts (portions/day)	Baseline	6.4 ± 3.1	6.4 ± 2.8	6.7 ± 3.2	7.1 ± 3.5	0.623	0.141
	Change at 1 month	0.1 ± 2.9	0.2 ± 2.2	1.1 ± 2.7	1.6 ± 2.5	0.328	< 0.001
	Change at 3 months	0.4 ± 2.7	0.3 ± 2.6	1.6 ± 3.4	1.3 ± 2.6	0.615	< 0.001
	Change at 6 months	− 0.1 ± 3.0	0.1 ± 2.5	0.6 ± 3.4	1.0 ± 2.9	0.413	0.024

^a^There are no significant interactions between the exercise and nutrition groups for all variables; ^b^Test for differences based on the exercise intervention status (average of the exercise group and the combination group compared with average of the control group and the nutrition group); ^c^Test for differences based on the nutrition intervention status (average of the nutrition group and the combination group compared with average of the control group and the exercise group); ^d^Change from baseline

The results of intervention effects on physical performance and mental health outcomes are summarized in Table 4. Significant interaction effects between the intervention groups and time were observed regarding handgrip strength (*p* = 0.004), upper body flexibility (*p* <  0.001), lower body flexibility (*p* = 0.037), and lower extremity strength (*p* <  0.001). Significant main effects observed in the intervention groups were regarding handgrip strength (*p* = 0.023), upper body flexibility (*p* = 0.020), lower body flexibility (*p* = 0.020), and lower extremity strength (*p* <  0.001). No statistically significant interaction effects were observed between the intervention groups and time, and the main effects of the intervention groups in 10-m gait speed. After the 6-month follow-up, compared with the control group, the beneficial improvements in handgrip strength were observed in the exercise (difference in handgrip strength change from baseline: 2.00; 95% CI: 1.16, 2.84; *p* <  0.001), nutrition (1.09; 95% CI: 0.26, 1.93; *p* = 0.011), and combination (1.30; 95% CI: 0.45, 2.14; *p* = 0.003) groups. Moreover, significant improvements in physical performance were observed in 10-m gait speed, upper body flexibility, lower body flexibility, and lower extremity strength in the exercise, nutrition, and combination intervention groups at 6 months.

**Table 4:** Intervention effects on physical performance and mental health outcomes during the 6-month study period

		Mean ± SD				Difference in difference: change from baseline in each intervention group minus change from baseline in control group (95% CI)			*p* for groups × time interaction	*p* for groups	*p* for time
Outcomes		Control	Exercise	Nutrition	Combination	Exercise	Nutrition	Combination			
Handgrip strength (kg)	*n*	80	79	82	77				0.004	0.023	< 0.001
	Baseline	25.0 ± 7.8	24.5 ± 7.7	24.9 ± 9.2	26.9 ± 8.5						
	1 month	25.1 ± 7.8	25.4 ± 8.6	24.9 ± 9.2	27.6 ± 8.4	0.91 (0.06, 1.75)	−0.05 (− 0.89, 0.78)	0.65 (− 0.19, 1.50)			
	3 months	25.1 ± 7.7	25.6 ± 8.8	25.8 ± 9.1	27.8 ± 8.5	1.10 (0.25, 1.94)^a^	0.83 (− 0.01, 1.66)	0.81 (− 0.04, 1.66)			
	6 months	25.3 ± 7.7	26.7 ± 8.7	26.3 ± 8.9	28.5 ± 8.7	2.00 (1.16, 2.84)^a^	1.09 (0.26, 1.93)^a^	1.30 (0.45, 2.14)^a^			
10-m gait speed (seconds)	*n*	80	79	83	77				0.159	0.226	< 0.001
	Baseline	10.7 ± 3.2	12.5 ± 7.7	13.0 ± 7.4	11.4 ± 4.3						
	1 month	10.6 ± 3.2	12.2 ± 8.1	12.4 ± 6.9	11.1 ± 4.0	−0.21 (− 0.78, 0.35)	− 0.48 (−1.04, 0.08)	− 0.20 (− 0.77, 0.37)			
	3 months	10.6 ± 3.3	12.3 ± 8.5	12.4 ± 6.8	10.9 ± 3.8	− 0.14 (− 0.70, 0.43)	− 0.56 (− 1.12, 0.003)	− 0.49 (− 1.06, 0.08)			
	6 months	10.8 ± 3.4	12.1 ± 8.1	12.3 ± 6.5	10.7 ± 3.4	−0.41 (− 0.98, 0.16)	− 0.81 (− 1.37, − 0.25)^a^	−0.81 (− 1.38, − 0.24)^a^			
Upper body flexibility (cm)	*n*	76	73	76	73				< 0.001	0.020	0.408
	Baseline	−13.9 ± 12.3	− 12.9 ± 16.7	−13.2 ± 12.0	−12.4 ± 12.5						
	1 month	−14.6 ± 12.2	− 13.1 ± 14.5	−13.1 ± 12.4	−11.4 ± 12.4	0.49 (− 1.62, 2.60)	0.82 (− 1.27, 2.90)	1.71 (−0.40, 3.82)			
	3 months	−15.8 ± 10.0	− 12.2 ± 13.3	−14.5 ± 10.0	−11.3 ± 13.3	2.54 (0.43, 4.65)	0.54 (− 1.55, 2.63)	3.07 (0.96, 5.18)^a^			
	6 months	−16.9 ± 10.5	−10.6 ± 12.7	−14.3 ± 10.3	−10.5 ± 12.5	5.21 (3.10, 7.31)^a^	1.86 (− 0.23, 3.94)	4.92 (2.81, 7.03)^a^			
Lower body flexibility (cm)	*n*	76	75	79	74				0.037	0.020	0.810
	Baseline	−4.6 ± 8.9	−2.9 ± 8.5	−5.2 ± 8.1	−3.3 ± 7.6						
	1 month	−5.5 ± 8.2	−2.1 ± 8.5	−5.4 ± 7.5	−3.0 ± 7.4	1.76 (0.39, 3.13)^a^	0.69 (−0.66, 2.05)	1.27 (−0.11, 2.65)			
	3 months	−5.5 ± 7.9	−2.0 ± 8.6	−5.6 ± 7.3	−2.2 ± 6.8	1.76 (0.39, 3.14)^a^	0.45 (−0.90, 1.81)	2.00 (0.63, 3.38)^a^			
	6 months	−5.8 ± 8.1	−1.8 ± 9.0	−6.0 ± 7.4	−2.3 ± 7.2	2.33 (0.96, 3.70)^a^	0.39 (−0.97, 1.74)	2.17 (0.79, 3.55)^a^			
Lower extremity strength (number)	*n*	69	61	70	64				< 0.001	< 0.001	< 0.001
	Baseline	1.6 ± 3.0	3.9 ± 5.2	2.0 ± 3.3	3.8 ± 4.3						
	1 month	1.8 ± 3.1	5.3 ± 6.0	2.1 ± 3.5	5.7 ± 6.0	1.25 (0.30, 2.21)^a^	−0.07 (−1.00, 0.85)	1.70 (0.76, 2.65)^a^			
	3 months	1.8 ± 3.0	5.8 ± 5.8	2.0 ± 3.4	6.9 ± 6.6	1.74 (0.78, 2.70)^a^	−0.12 (−1.04, 0.81)	2.93 (1.99, 3.88)^a^			
	6 months	1.7 ± 2.9	6.9 ± 6.7	2.4 ± 3.5	8.0 ± 7.0	2.95 (1.99, 3.90)^a^	0.34 (−0.58, 1.26)	4.05 (3.11, 5.00)^a^			
GDS (scores)	*n*	80	79	83	77				0.497	0.343	0.255
	Baseline	2.2 ± 1.7	2.5 ± 1.8	2.6 ± 1.9	2.4 ± 1.6						
	1 month	2.4 ± 1.9	2.5 ± 1.7	2.6 ± 2.0	2.3 ± 1.4	−0.19 (−0.62, 0.25)	−0.20 (− 0.63, 0.23)	− 0.32 (− 0.76, 0.12)			
	3 months	2.5 ± 1.8	2.6 ± 1.7	2.4 ± 1.8	2.3 ± 1.6	− 0.15 (− 0.58, 0.29)	−0.45 (− 0.89, − 0.02)	−0.37 (− 0.81, 0.07)			
	6 months	2.4 ± 1.7	2.3 ± 1.6	2.4 ± 1.9	2.2 ± 1.6	−0.43 (− 0.86, 0.01)	− 0.42 (− 0.85, 0.01)	−0.38 (− 0.82, 0.06)			
SF-12 MCS (scores)	*n*	80	79	83	76				0.567	0.624	< 0.001
	Baseline	53.8 ± 7.9	53.9 ± 8.1	53.4 ± 7.8	55.8 ± 7.0						
	1 month	54.5 ± 7.7	54.4 ± 8.3	53.9 ± 8.0	56.3 ± 6.7	−0.22 (−1.87, 1.43)	−0.24 (−1.87, 1.39)	− 0.22 (− 1.89, 1.44)			
	3 months	54.0 ± 7.7	55.2 ± 7.9	55.0 ± 6.1	57.0 ± 6.3	1.09 (− 0.56, 2.74)	1.33 (− 0.30, 2.96)	0.94 (− 0.73, 2.60)			
	6 months	54.3 ± 7.1	55.9 ± 6.9	56.0 ± 5.9	57.3 ± 6.6	1.58 (−0.07, 3.23)	2.12 (0.49, 3.75)^a^	1.05 (−0.61, 2.72)			

*CI* confidence interval; *GDS* Geriatric Depression Scale; *SF-12 MCS* 12-Item Short Form Health Survey mental component summary; ^a^Pairwise comparisons (interventions vs. control) were significant at the post hoc significance level of 0.017

In the mental health outcomes, significant interaction effects were not seen between the intervention groups and time, and the main effects of the intervention groups in GDS and SF-12 MCS. At the 6-month follow-up, the SF-12 MCS score change from baseline was significantly greater in the nutrition group than in the control group (difference in score change from baseline: 2.12; 95% CI: 0.49, 3.75; *p* = 0.011) (Table 4).

## Discussion

In this study, we randomized the pre-frail or frail older adults into four designated groups — exercise, nutrition, combination, and control — to perform a 3-month individualized intervention and 3-month self-maintenance program at home. To the best of our knowledge, this study is the first randomized controlled trial to assess the effects of individualized home-based exercise and nutrition interventions on frailty management for the pre-frail or frail older adults. Our results support the hypothesis that an individualized home-based interventions program ameliorate frailty and improve physical and mental health outcomes for pre-frail or frail older adults. Our program was a genuinely home-based intervention because the personal contact between the participants and professionals lasted for only 1 h for a baseline evaluation, 5 min on the third day, 5 min at the end of the second month for a telephone greeting, and 30 min at the 1-month and 3-month follow-ups for reassessment and prescription revision. The cost of this home-based intervention program was considerably low, and the 3-month program costs per participant (including expenditures for program materials and professional time) were USD 29, USD 57–78, and USD 81–100 for those in the exercise, nutrition, and combination groups, respectively. Another innovative approach was the use of inspirational cards to increase participants’ compliance. The novelty of this intervention program was to translate a professional prescription of individualized physical and nutritional intervention into a handy program that could be effectively carried out by community-dwelling older adults at home.

Previous studies have shown that physical exercise helps older adults increase muscle strength [28, 29], increase brain volume (gray and white matter regions) [30], and help them to prevent falls [31]. Moreover, physical exercise improve the mobility and physical functioning of older adults with mobility problems, physical disability, or multiple morbidities [32]. The effects of exercise training have been validated for older adults, irrespective of whether they are in robust condition or with functional impairment. Notably, frail older adults are at high risk for multiple comorbidities and adverse events. However, intervention studies to improve the frail conditions of pre-frail or frail older adults remain limited [6, 7, 10, 33]. This study not only recruited pre-frail or frail older adults based on well-defined criteria but also evaluated the efficacy of individualized home-based exercise and nutrition interventions for improving frailty syndrome.

Notably, nutrition interventions for the older population have been widely proposed. Some have demonstrated the efficacy for improving adverse outcomes, such as reduction of the incidence of malnutrition [34, 35] and type 2 diabetes [36]. Some studies revealed that nutritional supplements increase the total energy intake and body weight [37], protein intake [38], and usual gait speed [39] in older adults who are at a risk of malnutrition. However, these studies did not investigate the nutritional effects on the improvement of frailty and related measurements. In this study, we demonstrated that our nutrition intervention (individualized dietary consultation plus food supplements) were effective even after food supplements were stopped and steered the dietary patterns of the participants towards the recommended diet with significant increments in the intakes of the three food groups (beans/fish/meat/eggs, dairy, and oils/nuts); this resulted in an increases in the total energy and percentage of calories from protein. Therefore, this significant shift in dietary pattern coincides with the observed positive effects such as reduced frailty scores, improved handgrip strength, and gait speed.

Most studies agree that multicomponent interventions are promising approaches to prevent functional decline and decrease the risk of disability in older adults. Previous results have demonstrated the effectiveness of multidomain interventions on improving cognitive function [40], health-related quality of life [41], and frailty [7]. These intervention programs were most often carried out through group sessions. However, older people are often not able to leave their home to attend such group-based programs, particularly in rural areas. Therefore, volunteer-based projects have been explored to enhance the outreach of public health care systems in disadvantaged communities [42]. This volunteer-assisted home-based intervention program was effective in improving the malnutrition and frailty. Nonetheless, this program employed trained nonprofessional volunteers to assist older subjects at home and implement the intervention for 3 months. However, such intervention efforts could have been compromised owing to insufficient professional expertise. Moreover, some study effects could be confounded because the volunteers in the control group might have delivered the exercise or nutrition intervention information because a standardized training program was carried out for all volunteers. Compared with previous intervention studies for improving frailty syndrome, our study demonstrates that home-based exercise and nutrition interventions prescribed by professional physiotherapists and dietitians are effective not only for self-management but also for improving frailty and physical performance in pre-frail or frail older adults.

We observed that our exercise and nutrition intervention groups had no remarkable effects on most of the mental health outcomes, except the nutrition intervention group that exhibited a positive effect with an improved SF-12 MCS score after intervention. A previous study had also supported the beneficial effect of a nutrient-dense protein-energy liquid supplement in conjunction with active encouragement to improve food intake on the emotional role functioning [37]. Two randomized controlled trials reported that exercise training could improve depression syndrome in older adults with depression [43, 44]. However, the current study observed no differences regarding GDS between the exercise and control groups. A potential explanation of this inconsistent result would be that our participants probably had healthier psychological status because we excluded those with GDS scores of ≥10. Therefore, additional studies should explore the effectiveness of home-based exercise and nutrition interventions on depression syndrome.

Nonetheless, some limitations of this study must be acknowledged. First, among the subjects who agreed to participate (*n* = 423), 84 older adults (19.86%) were excluded based on the exclusion criteria. Despite the small effects of self-selection in our study, it may affect the generalizability of our results. In addition, the generalizability of our intervention results may not apply to older adults who are not frail or have conditions listed in our exclusion criteria. From the viewpoint of frailty prevention, the effects of individualized home-based exercise and nutrition interventions require further investigation among older adults under robust conditions. Second, the 3-month intervention period was relatively short. The reduction in the frailty score between the interventions and control groups was not significantly different at the end of the third month. Instead, the effect became significant after the extended 3-month self-maintenance period when the participants in the intervention groups (exercise, nutrition, or combination) continued with self-exercise training and dietary patterns according to their designated intervention schedules. The continuing improvements in physical performance and dietary intake at 6 months could support these results. Third, we primarily showed changes in macronutrient distribution but did not calculate and compare changes in vitamin and mineral density of participants in the four groups because participants used certain commercial products for which the micronutrient information was unavailable. Nonetheless, we did report substantial dietary pattern changes in the groups receiving the dietary intervention (i.e., significant increases in the four food groups: dairy, protein foods, nuts or seeds, and fruits). Fourth, this study could not be implemented as a double-blind design; however, the outcomes measured by case managers complied with a blind assessment. Furthermore, the predetermined study purpose of this research was to evaluate the efficacy of individualized home-based exercise and nutrition interventions on overall frailty; therefore, we did not investigate each indicator of frailty separately. Consequently, we may not discern which aspect of frailty improved by the interventions. Finally, because the exercise and nutrition advice was customized per participants’ capability, it may be difficult to replicate the advice. Moreover, even if replicated, it may be hard to pinpoint whether an inconsistent result is due to professionals’ advice or some other factor.

## Conclusions

This study demonstrates that individualized home-based exercise and nutrition interventions are effective in improving frailty and physical performance among community-dwelling pre-frail or frail older adults by changing their dietary intake patterns and engaging them in exercise training. Moreover, nutrition intervention may be helpful to improve the mental health in pre-frail or frail older adults. The health promotion agencies should, therefore, identify pre-frail or frail older adults from community-based hospitals, clinics, or public health stations and then implement an individualized exercise and nutrition intervention program to improve and manage frailty.

## Data Availability

The datasets used in the current study are not publicly available due to the legal restrictions of Personal Information Protection Act legislated by the government of Taiwan. Data are available from corresponding authors upon reasonable request with valid proposals and confidentiality agreement.

## Appendix

### Method to estimate the servings of the six food groups

We grouped all the foods consumed to six food groups and estimated the servings of each group by its content of carbohydrate, protein, or calorie as listed in the following table.

**Table:**

Food groups	Definition of one serving
Cereal, grain, tuber	15 g of carbohydrate
Bean, fish, egg, poultry, meat	7 g of protein
Dairy	8 g of protein
Oil, nut, seed	5 g of fat
Vegetable	25 cal
Fruit	60 cal

## Abbreviations

ADL: Activities of daily living
BMI: Body mass index
CI: Confidence interval
GDS: Geriatric Depression Scale
IPAQ: International Physical Activity Questionnaire
ITT: intention-to-treat
MMSE: Mini-Mental State Examination
SF-12 MCS: 12-Item Short Form Health Survey mental component summary
