# Correction to: Individualized home-based exercise and nutrition interventions improve frailty in older adults: a randomized controlled trial

**DOI:** 10.1186/s12966-019-0905-3

**Published:** 2019-12-23

**Authors:** Tsung-Jen Hsieh, Shin-Chang Su, Chun-Wei Chen, Yaw-Wen Kang, Ming-Hsia Hu, Li-Lin Hsu, Szu-Yun Wu, Likwang Chen, Hsing-Yi Chang, Shao-Yuan Chuang, Wen-Harn Pan, Chih-Cheng Hsu

**Affiliations:** 1 Institute of Population Health Sciences, National Health Research Institutes, 35 Keyan Road, Zhunan, Miaoli County 35053 Taiwan; 2 School and Graduate, Institute of Physical Therapy, College of Medicine, National Taiwan University, Taipei, Taiwan; 3 Department of Chest Medicine, Miaoli General Hospital, Miaoli, Taiwan; 4 Department of Physical Medicine and Rehabilitation, Miaoli General Hospital, Miaoli, Taiwan; 5 Food Industry Research and Development Institute, Hsinchu, Taiwan; 6 Institute of Biomedical Sciences, Academia Sinica, 128 Academia Road, Section 2, Nankang, Taipei 11529 Taiwan; 7 Department of Health Services Administration, China Medical University, Taichung, Taiwan; 8 Department of Family Medicine, Min-Sheng General Hospital, Taoyuan, Taiwan

**Correction to: Int J Behav Nutr Phys Act (2019) 16:119**

**https://doi.org/10.1186/s12966-019-0855-9**

Following publication of the original article [1], the author reported that an abbreviation was incorrect in the original article;

1. In the Appendix the abbreviation ‘cal’ was incorrect. The correct abbreviation is ‘kcal’.
